# Leadership of the ultimate interdisciplinary team: Rehabilitation science at NIH

**DOI:** 10.1186/s12984-020-00696-0

**Published:** 2020-05-29

**Authors:** Alison N. Cernich

**Affiliations:** Eunice Kennedy Shriver National Institute of Child Health and Human Development, 31 Center Drive, Bethesda, MD 20892 USA

**Keywords:** Rehabilitation, NIH, Basic science, Clinical trials, Technology

## Abstract

**Background:**

Support for rehabilitation research at the National Institutes of Health (NIH) is robust and evolving. Since the time of its Blue Ribbon Panel on Rehabilitation Research, NIH has participated in several initiatives to coordinate the science and advance the field.

**Discussion:**

Collaborative teams must continue to address key limitations in the field, including the desire for broad application of rehabilitation interventions, the need for basic science and translational research, the support of clinical trials and standard approaches, and the integration of technology.

**Conclusion:**

Rehabilitation medicine is poised for critical advancements if interdisciplinary teams continue to work collaboratively to understand and address the needs of people with temporary or permanent functional limitations.

## Background

In 2012, the National Institutes of Health (NIH) convened a Blue Ribbon Panel on Rehabilitation Research to advise the NIH Director on the changes needed in the coordination and funding of rehabilitation science, the necessary training and research infrastructure for the field, and the methods by which training of the next generation of scientists in this area could be encouraged [1]. After the publication of this report in 2014, a new agenda at NIH was formed to act on the recommendations of the panel. It was in this climate that I began my tenure as the Director of the National Center for Medical Rehabilitation Research (NCMRR), part of NIH’s *Eunice Kennedy Shriver* National Institute of Child Health and Human Development (NICHD), in 2015. It was an honor and a privilege to lead efforts at NIH, and none of the work performed over the ensuing years would be possible without the staff of the Center, our NIH colleagues, our federal partners, and an involved and engaged community of researchers, stakeholders, and advocates. This was truly an interdisciplinary team that evolved the guidance and direction of rehabilitation research funded by NIH; we achieved many goals. In this commentary, I will provide a review of the past four and a half years of change at the NIH with respect to rehabilitation research, the programs initiated by the NIH rehabilitation research community, what is needed to evolve the science of rehabilitation, and on the challenges we continue to face.

### Leading change in Rehabilitation

The Blue Ribbon Panel served as a catalyst for the community, and NIH recognized the need for a robust and coordinated response to the recommendations. First and foremost, a research agenda with clear priorities was required by legislation. But more than simply meeting a requirement, the Research Plan on Rehabilitation [2] allowed NIH and the rehabilitation community to establish cross-cutting priorities that would serve the science. This effort highlighted the need not only for clinical research, which was prominent in the NIH portfolio, but also the contributions of basic and translational science. The NIH community recognized that rehabilitation is vital for persons with many different diseases and conditions and took a disease agnostic approach to the development of priorities.. This disease-agnostic approach provided the basis for the plan and enabled NIH to specify priorities applicable across its Institutes and Centers. It also allowed for the integration of rehabilitation research concepts into multiple funding opportunities, workshops, and research efforts.

The scientific conference that supported the development of the research plan highlighted the key tenet that rehabilitation is applicable across diseases and conditions [3]. It also brought forth an energy and sense of collaboration across disciplines and an understanding that all of NIH was engaged in the rehabilitation mission, not just NCMRR. This concept that rehabilitation research is an NIH-wide mission is critical to the continued growth of the field. The investigators in the field are now recognizing that many Institutes and Centers across NIH are receptive and willing to fund science in this area because of the broad dissemination of this message by all engaged Institutes and Centers. The NIH community feels this is the main driver for the nearly $175 million increase in the NIH rehabilitation research portfolio over the last 5 years [4].

After unifying the rehabilitation community at NIH with a set of shared priorities, disseminating the willingness for the NIH to support rehabilitation research across the Institutes and Centers through the conference and outreach at professional meetings, and regular communication with the field regarding research opportunities through communication efforts, there was a sense that the rehabilitation community could accomplish a great deal.

The Medical Rehabilitation Coordinating Committee, which is a coordinating committee of 20 Institutes and Centers within NIH, began to pursue multiple lines of effort to further the work stemming from the Research Plan and the conference that are yielding results now [5]. This encompassed collaborations within the NIH, integration of rehabilitation into trans-NIH efforts, and work across federal agencies to better address the priorities and gaps in the rehabilitation research agenda.

Within NIH, the NCMRR, the National Institute of Neurological Disorders and Stroke (NINDS), and the Office of Disease Prevention will host a conference on the benefits of physical activity for individuals who use wheelchairs, with an accompanying systematic review completed by the Agency on Healthcare Research and Quality to support the inclusion of evidence-based recommendations in federal guidelines. NCMRR recently launched a Common Data Elements effort for rehabilitation in collaboration with the NINDS. The NIH community continues to sponsor funding announcements, most notably in community-based cardiac rehabilitation, rehabilitation research infrastructure, and training and career development.

The NIH Medical Rehabilitation Coordinating Committee was integrally involved in large NIH efforts, such as the All of Us program, where we advocated for the inclusion of individuals with disability, successfully including a community partner for the program and establishing the evidence for the participant-provided information survey for disability [6]. We engaged with the NIH BRAIN initiative and successfully advocated for work that promoted human trials for brain-computer interfaces that promote function for individuals with severe disability, as well as neural prosthetics and new approaches to deep brain stimulation [7]. We worked with colleagues to promote rehabilitation across the Helping to End Addiction Long-term^SM^ Initiative, or NIH HEAL Initiative^SM^, and were successful in gaining support for multiple applications, including those focused on lower back pain, cancer rehabilitation, primary care provision of physical therapy, and new devices to treat or remediate pain [8].

NIH is consistently working with federal partners to advance the coordination of rehabilitation research efforts. The NCMRR funded an effort to advance the field of limb amputation and limb salvage by supporting the development of a registry in collaboration with the Department of Defense, that is moving toward an initial pilot in spring of 2020. NCMRR and colleagues from the National Institute of Diabetes and Digestive and Kidney Diseases are working with our federal colleagues from the Departments of Defense, Veterans Affairs, and throughout the Department of Health and Human Services to standardize common data elements in the limb loss field. This work to arrive at common definitions and standards across agencies, we hope, will serve as an exemplar to ease regulatory and reimbursement considerations for new approaches and devices for this population. NCMRR collaborated with the Centers for Disease Control and Prevention to fund the integration of disability questions into a national pregnancy surveillance effort to support evidence development for obstetric care for individuals with disability. NCMRR, the National Institute on Deafness and Other Communication Disorders and the Fogarty International Center worked collaboratively to support efforts on global implementation of rehabilitation with the US Agency for International Development and the World Health Organization and helped to establish global priorities for rehabilitation research, data, and infrastructure.

Recent NIH-sponsored workshops and conferences have focused on innovative technologies for pediatric rehabilitation, clinical trials in rehabilitation, strategic directions for cerebral palsy and spinal cord injury research, integrative treatments, and medical device development and commercialization. The NIH Medical Rehabilitation Research Coordinating Committee is an engaged team, ready to pursue any avenue to promote rehabilitation as a critical area of science.

### Evolution and direction of the field: debunking dogma

As the field continues to evolve, I encounter several recurring themes when I attend conferences, workshops, meetings, and gatherings that occur in scientific, legislative, advocacy, and stakeholder domains. From my perspective, these themes can hamper progress, discourage young investigators from adopting new approaches, and limit innovation. I will highlight and rebut a few of these in the hope that the field can change the dogma.

*“Rehabilitation is intended only for people with chronic disability.”* While it is true that individuals with chronic disability require evidence to support their need for continued access to rehabilitation over the lifespan, this should not be the only goal in our field. Rehabilitation and the science underlying rehabilitation approaches are critical to remediating temporary functional limitations from injury or surgery, to enable improved function after treatment for disease (e.g., cancer), and to engage older individuals who are on the cusp of aging into functional limitations. Imposing limits or curtailing a broader agenda limits the field and does not allow for broader applications.

*“Basic science is not a priority for rehabilitation research.”* The basic science portfolio for rehabilitation, unlike many portfolios at NIH, is relatively small. However, concentrating only on interventions and outcomes misses a fundamental underpinning of science that contributes to advances that are well-known in other fields. The discovery of biomarkers, quantifiable variables that can be monitored, is critical to prognosis, diagnosis, and monitoring of recovery across conditions. Moreover, these biologic, imaging, or sensor-based measures allow for the development of interventions that are more precisely targeted to symptoms or individuals. The field is critically lacking endeavors in genomics, epigenomics, proteomics, transcriptomics, the microbiome, serum-based biomarkers, and imaging indicators that could contribute to more evidence-based care. This involves a continuum of research that is underemphasized in rehabilitation medicine and requires new partnerships with scientists in these fields to advance more quickly.

*“We cannot conduct large, adequately powered trials of rehabilitation interventions.”* After working as a mental health clinician and researcher in the Department of Veterans Affairs, where psychotherapeutic interventions were randomized, tested, implemented, revised, and evaluated [9], there is no question that rehabilitation could mirror this approach. What interferes with progress in this area are two commonly accompanying statements: *“Each patient is different, you can’t have a standard approach,”* and *“There is no way to blind or control for exposure to the treatment.”* Work currently ongoing in the field rebuts these contentions [10]. There is a glaring need for standardization of approaches to support the rigor and reproducibility of the science. Standardization does not ignore the individual, it allows for broader application of techniques supported by evidence. New approaches to clinical trials, such as adaptive trials, pragmatic trials, and other methodologic approaches, may allow for more flexibility in clinical trial design and can be used to evaluate whether an intervention or approach is effective in the real world.

*“It is hard to quantify rehabilitation, identify treatment targets, and measure progress”.* Technology holds enormous promise for quantitative measurement of movement, discovery of biomarkers through high-throughput analyses, screening for potential treatment targets, outcome measurement, and monitoring social participation and community engagement. While technology is not the only solution for these challenges in rehabilitation research, the field needs to consider how to move to more rigorous and quantifiable metrics. Integration of sensors, videocapture, app-based tools, gaze technologies, voice analysis, new imaging approaches, innovative modeling, and machine learning approaches are critical to supporting the science and to engaging young investigators. Commercial off-the-shelf tools and research kits are available and the costs are lowering quickly for tools that were once prohibitively expensive. Collaborations are a key part of this work; technology companies, medical equipment providers, computer scientists, and engineers are increasingly interested in how their skills and abilities can assist individuals with temporary or chronic functional limitations. This is not to say that technology is the only solution to this set of problems, and there is a clear need to ensure that the solutions are cost-effective and accessible. However, the field of rehabilitation will miss opportunities and advancements if we do not continue to progress in this area.

## Conclusion

Leading the National Center for Medical Rehabilitation Research was an honor and a privilege. In this position, I had the opportunity to oversee new and emerging science, promote and support the field, and meet people all over the world who see rehabilitation as a global need, critical to enabling health. At every meeting or conference, there is a young investigator with passion, drive, and a sense of novelty that inspires others to keep advocating for rehabilitation.

Rehabilitation is my professional mission. Throughout my career, starting with my undergraduate training, I found many ways to engage in the work. I came into this leadership role after serving in roles with individuals with chronic or temporary disability, from driving the van to the grocery and assisting with shopping, to managing a polytrauma care clinic, to conducting research on the effect of exercise on cognitive recovery. The one tenet that followed me throughout is “keep the person in the center.” My hope is that the next generation of basic scientists understands this tenet as well as the clinician scientist. Scientific work divorced from an end goal of improving health and optimizing function for a person can be an empty endeavor.

There is an African proverb that reads “If you want to go fast, go alone; if you want to go far, go together.” In rehabilitation, different than some other disciplines, the work is fundamentally team-based. Throughout my career, I was honored to work with people contending with functional limitations, rehabilitation teams, interdisciplinary research collaborators, and my federal colleagues to advance a rehabilitation treatment plan, research project, or large policy or research agenda. The focus was to improve function, promote participation, and ensure that individuals with limitations are included in scientific research. Finding methods to recognize the efforts of these teams, especially in light of the challenges team science poses to investigators seeking career advancement, will be critical in the future. This will require effort to incorporate collaboration metrics rather than individual effort in evaluation and to de-emphasize traditional measures of success in favor of those that value the efforts that underly collaboration (e.g., number of multiple primary investigator awards vs. primary investigator). I hope that the community continues to see the value in this team-based approach to overcome the challenges facing our field and to advance the science. Rehabilitation is critical to enabling healthy and optimal lives, and together, we will continue to support the evidence needed to achieve that goal.

## Data Availability

Not applicable.

## Abbreviations

NIH: National Institutes of Health
NCMRR: National Center for Medical Rehabilitation Research
NICHD: *Eunice Kennedy Shriver* National Institute of Child Health and Human Development
BRAIN: Brain Research through Advancing Innovative Neurotechnologies Initiative®
NIH HEAL: Helping to End Addiction Long-term^SM^ Initiative
